# Composite Hydrogel Dressing with Drug-Release Capability and Enhanced Mechanical Performance

**DOI:** 10.1021/acs.biomac.5c00505

**Published:** 2025-08-12

**Authors:** Jie Yang, Fanlei Yang, Wenhao Xu, Xiuqin Yu, Zhaozhu Zheng, Xiaoqin Wang, Kaili Chen, Jia Yu, Gang Li

**Affiliations:** † National Engineering Laboratory for Modern Silk, College of Textile and Clothing Engineering, Soochow University, Suzhou 215123, China; ‡ Orthopedic Institute, Department of Orthopaedic Surgery, The First Affiliated Hospital, Suzhou Medical College, Soochow University, Suzhou 215006, China; § Department of Dermatology, the First Affiliated Hospital of Soochow University, Suzhou 215006, China; ∥ Department of Materials, Imperial College London, Exhibition Road, London SW7 2AZ, U.K.; ⊥ Botnar Research Center, University of Oxford, Old Road, Headington, Oxford OX3 7LD, U.K.; # School of Physical Education and Sports, Soochow University, Suzhou 215006, China

## Abstract

Wound care is a critical concern due to the rising prevalence of chronic and infected wounds, which demands improved treatment solutions. Conventional dressings often fall short in delivering effective drug release and mechanical performance, limiting their clinical utility. In this study, we present a composite hydrogel dressing incorporating berberine-loaded silk fibroin microspheres and *Calotropis gigantea* fibers (CGFs). This innovative dressing achieves sustained drug release and enhanced mechanical strength. The hydrogel exhibits excellent antibacterial activity (97.40 ± 0.53%) against *Staphylococcus aureus*, efficient drug loading, and prolonged release profiles. The addition of CGFs significantly enhances mechanical performance (maximum compressive strength of 295.74 ± 27.45 kPa), hemostatic efficiency (0.33%), and adaptability to dynamic wound environments. Our composite hydrogel dressing demonstrates superior biocompatibility and accelerates wound healing by promoting neovascularization, hair follicle regeneration, and collagen deposition. This multifunctional solution offers a promising advancement in next-generation wound care technologies.

## Graphical abstract

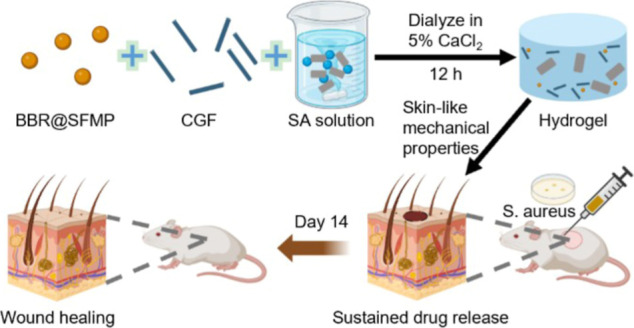

## 1 Introduction

Wound management plays a crucial role in healthcare, as untreated wounds can lead to complications such as infections, chronic inflammation, delayed healing, and scarring.^1–3^ Burns, abrasions, and surgical wounds affect millions of people annually, driving the demand for innovative wound care materials.^4,5^ Traditional dressings, like cotton gauze, are affordable and widely used but often dry out and adhere to wounds, causing pain during removal and the disruption of the healing process.^6^ In contrast, state-of-the-art wound dressings are designed to maintain a moist environment, which significantly accelerates healing.^7^ Previous research demonstrated that wounds kept in a moist environment heal twice as quickly as those exposed to air, inspiring the development of hydrogel-based dressings that retain moisture, reduce infections, and promote tissue regeneration. Among various hydrogel materials, sodium alginate (SA) stands out for its excellent biocompatibility, inherent hydrophilicity facilitating moisture retention crucial for wound healing, and its ability to form hydrogels under mild conditions (e.g., ionic cross-linking with Ca^2+^), making it a widely used base material for wound dressings.^8^

Despite these advancements, many current hydrogel dressings face critical limitations, particularly in drug delivery and mechanical strength. The rapid release of drugs and short therapeutic durations necessitate frequent dressing changes, reducing drug utilization efficiency and impeding optimal healing. Furthermore, the insufficient mechanical strength of many hydrogel dressings increases the risk of tearing or deformation, restricting their use in dynamic environments and compromising patient care.^9^ Recent advances in smart-responsive hydrogels, such as glucose/pH dual-responsive systems, offer promising strategies to achieve on-demand drug release by leveraging pathological microenvironment cues.^10^ Currently, hydrogels incorporating antimicrobial, anti-inflammatory, and osteogenic therapies have demonstrated enhanced efficacy in addressing the multifactorial pathological process of periodontitis. The composite hydrogel developed in this study builds upon this concept by combining multiple materials to achieve sustained drug release and enhanced mechanical properties.^11^ As a natural plant fiber, *Calotropis gigantea* fiber (CGF) contains natural latex component which imparts pro-coagulant, antibacterial and anti-inflammatory properties. Its high cellulose content (60–70%) and lignin-based structure, along with an 80–90% hollow pore structure, enhance the mechanical strength of the hydrogel through hydrophilic interactions and facilitate exudate absorption *via* the capillary effect. These synergistically features accelerate the hemostasis, achieving simultaneous optimization of bioactivity and mechanical properties, thereby providing multifunctional support for the wound dressing.^12,13^ Addressing these challenges requires the development of innovative material combinations. Silk fibroin (SF), a natural protein derived from silk, offers remarkable advantages including outstanding biocompatibility, biodegradability and tunable mechanical properties. Moreover, SF can be engineered into various drug delivery system, such as microspheres, for sustained release. Its inherent β-sheet structure further enhances the structural stability of these carriers.^14^ Addressing these challenges through the use of such materials is essential for developing wound dressings that integrate sustained drug release with enhanced mechanical properties. These advancements have the potential to improve patient comfort, safety, and therapeutic efficiency, ultimately offering a durable and effective solution for advanced wound management.

Herein, this study presents a composite hydrogel dressing that integrates long-lasting drug release with superior mechanical properties. Berberine (BBR)-loaded silk fibroin microspheres (BBR@SFMPs), prepared *via* a “two-step method”, and CGFs were incorporated into a SA hydrogel, forming the SA-BBR@SFMP-CGF. The microspheres significantly prolong BBR release while achieving effective antibacterial concentrations within 1 h. Meanwhile, the inclusion of CGFs enhances the hydrogel’s resistance to deformation, yielding comparable to that of human skin. In a full-thickness skin wound model infected with *Staphylococcus aureus* , the composite hydrogel demonstrated potent antibacterial efficacy and promoted neovascularization, follicular proliferation, and collagen deposition, thereby accelerating wound healing. By overcoming the limitations of traditional wound dressings, this hydrogel offers a multifunctional platform that combines prolonged antibacterial efficacy with improved mechanical performance, providing a robust solution for dynamic wound environments and expanding the biomedical applications of CGFs (Table 1).

**Table 1:** Comprehensive Performance Benchmarking (N.R.: Not Reported)

category	metric	this work	commercial alginate dressing	SA-GEL hydrogel^15^	SF microspheres^16^
physical properties	compression modulus	30.30 kPa	15–20 kPa	25 kPa	N.R.
	swelling ratio	411.80%	500–600%	350%	N.R.
antibacterial performance	anti-S. aureus rate	97.40%	>90% (silver dressing)	N.R.	>99%
	release duration	>15 D	none	7 D	14 D
biological performance	healing rate at Day 7	66.13%	50–60%	58% (noninfected)	70% (noninfected)
	neovascularization density (day 7)	16.76%	N.R.	12%	18%
safety	hemolysis rate	0.33%	<5%	<2%	1.2%
	cell viability (day 5)	106.71%	N.R.	>95%	>98%

## 2 Experimental Section

### 2.1 Materials and Reagents

Silkworm cocoons were purchased from Zhejiang Shengzhou Xiehe Silk Co. Ltd. Anhydrous sodium carbonate (Na_2_CO_3_), berberine (BBR), calcium chloride (CaCl_2_), and PBS powder were purchased from Shanghai Sinopharm Group Chemical Reagent Co. Ltd. Trypsin (bovine source) was sourced from Aladdin Reagent (Shanghai), and dialysis bags were purchased from Spectrum Medicine (USA).

### 2.2 Preparation Process for SF Solution

Silk was boiled in deionized water with anhydrous Na_2_CO_3_ for 30 min to remove sericin, followed by rinsing thoroughly with deionized water and drying out overnight. The dried silk was dissolved in a lithium bromide solution at 60 °C for 4 h. The solution was dialyzed against deionized water for 36 h and then filtered. A dish (*m*_1_) was preweighed for measuring the SF solution concentration. One mL of SF solution was added to the dish, and measured as the total mass (*m*_2_). After drying, the dish was weighed again (*m*_3_). The mass fraction (wt %) was calculated using the below formula

$$wt\;\left(\%\right)=\frac{m_{3}-m_{1}}{m_{2}-m_{1}}\times 100\%$$

### 2.3 Preparation of BBR@SFMP

SF solutions were prepared in different concentration varying from 1 wt %, 5 wt %, to 10 wt %. Each solution was mixed in potassium phosphate solution (1.25 M, pH 8) at a volume ratio of 1:5 (V/V).^17^ The mixture was left to stand at 4 °C for 2 h and then at room temperature for 12 h. Centrifugation (8000 rpm, 10 min) was used for purification, by discarding the supernatant and the precipitate was resuspended in deionized water for another twice wash cycles ultrasonicated (35% power, 3 min). The final SFMP suspension was freeze-dried at −80 °C for 48 h to obtain powdered SFMPs.

Saturated BBR solution (2 mg/mL) and SFMPs (2 mg/mL) were ultrasonicated at 35% power for 3 min separately and mixed at a mass ratio of 1:1. The mixture was incubated overnight in the dark. The supernatant was centrifuged (8000 rpm, 10 min) and collected for further testing, while the precipitate was resuspended in deionized water using ultrasonication (35% power for 3 min). This process was repeated three times to ensure thorough suspension. The resuspended sample was freeze-dried in the same manner to produce BBR@SFMP.

### 2.4 Particle Size and Zeta Potential Testing

Characterization of the particle size and potential was performed at room temperature using a Zetasizer Particle Sizer (Nano ZS90). The microsphere powder (0.1 mg/mL) was dissolved in deionized water by sonication forming a microsphere suspension. One mL of suspension was added to the particle size test dish and the potential test dish for the measurement.^18^

### 2.5 Microsphere Drug Loading Content (DLC)

The absorbance values of different concentrations of BBR solutions were detected at 345 nm using an enzyme labeling instrument (Synergy H1). The scatter plots of absorbance values versus concentration were plotted, and the linear regression equations were fitted.

The absorbance of the supernatant after centrifugation of the BBR@SFMP was detected, and the content of BBR m_2_ was determined according to the standard curve of BBR absorbance–concentration. The DLC was determined using the following formula

$$DLC\;\left(\%\right)=\frac{m_{1}-m_{2}}{m+m_{1}-m_{2}}\times 100\%$$

where m is the mass of the microspheres, *m*_1_ is the initial mass of the BBR used and *m*_2_ is the amount of BBR in the supernatant.

### 2.6 Preparation of Composite Hydrogel Dressing

A 2 wt % SA solution was prepared. Raw CGFs were prepared in ethanol for 5 min, followed by ultrasonically cleaning for 1 h with three wash cycles with deionized water and ethanol, which were subsequently air-dried in a fume hood to obtain processed CGFs. BBR@SFMPs and CGFs were separately dispersed in deionized water and then mixed in the SA solution at a 1:1 ratio. The mixture was dialyzed using CaCl_2_ solution for 12 h to form hydrogels.

The hydrogel samples were prefrozen in liquid nitrogen and freeze-dried for 48 h. The dried samples were stored in sealed containers for further use.

### 2.7 Morphology

The surface morphology of samples was examined using scanning electron microscopy (SEM) (Hitachi S-4800). Samples were mounted on conductive adhesive, sputter-coated (10 mA, 90 s), and imaged using SEM (5 kV, 10 μA). Porosity and pore size of composite hydrogels were obtained from SEM images analyzed by ImageJ.

### 2.8 Infrared Spectrum

The freeze-dried samples were scanned by an infrared spectrometer (Nicolet 5700) at a wavenumber of 400–4000 cm^–1^, and the secondary structure content of SF was obtained by Peakfit.

### 2.9 Mechanical Performance

Wet hydrogel samples were molded into cylinders (5 mm height × 10 mm diameter). Compression testing was performed with a texture analyzer (TMS-PRO) at a rate of 30 mm/min and 80% strain. Each sample was tested at least five times, with average value recorded. The hydrogel with the optimal performance was selected for a 50-cycle compression fatigue test. Cyclic compression tests for the best performing hydrogels were performed at fixed strains of 20%, 40%, and 60% for 50 cycles.^19^

### 2.10 Swelling Properties

The dry hydrogel sample (mass *W*_1_) was immersed in PBS (pH 7.4 and pH 5.5). At 1, 3, 6, 12, and 24 h, the sample was removed, blotted to remove surface liquid, and weighed (*W*_*t*_).^20^ The swelling ratio (SR) was calculated using the following formula

$$SR\;\left(\%\right)=\frac{W_{t}-W_{1}}{W_{1}}\times 100\%$$

Here, *W*_*t*_ – W_1_ represents the mass increase, and *W*_1_ is the initial dry weight.

### 2.11 In Vitro Degradation Performance

The dry weight of the hydrogel (*W*_0_) was recorded, and samples (20 mg/mL) were immersed in PBS (pH 7.4 and pH 5.5) containing 50 μg/mL trypsin. A control group without trypsin was included to evaluate its effect on degradation. Twelve groups with three independent replicates were prepared. Samples were incubated in sealed centrifuge tubes on a shaker at 37 °C with PBS solution exchanged at each sampling point.

At 1, 3, 6, 12, 24, 48, 96, and 144 h, hydrogels were taken out for rinsing with deionized water and dried at 60 °C for weight measurement (*W*_*t*_). The mass remaining ratio (MR) was calculated according to the formula

$$MR\;\left(\%\right)=\frac{W_{t}}{W_{0}}\times 100\%$$

### 2.12 In Vitro Release of BBR

The same steps as DLC were followed, and the absorbance–concentration standard curve of BBR in PBS (10 mmol/L, pH 7.4, 5.5) was established. Sample solutions were prepared in centrifuge tubes (hydrogel: 20 mg/mL, BBR@SFMP: 2 mg/mL) and placed in a thermostatic oscillator. At specific time points (1, 3, 6, 9, 12, 24, 48, 72, 96, 120, 240, 360 h), the tubes were centrifuged to collect the supernatant and change the liquid. The absorbance of the supernatant (release solution) was measured using an enzyme labeling instrument. The absorbance of the release solution was detected using an enzyme labeling instrument, and the content of BBR in the release solution was calculated according to the standard curve of absorbance–concentration.

The cumulative release rate (CRR) of BBR was determined using the below formula

$$CRR\;\left(\%\right)=\frac{C_{n}{\times V}_{0}+\left(C_{1}+C_{2}+...+C_{n-1}\right)\times V}{W\times X}\times 100\%$$

Here, *C*_*n*_ is the concentration at the nth sampling point; *V*_0_ is the volume of the release medium; *V* is the volume of each sample; *W* is the total mass of the sample; and *X* is the DLC (%).

### 2.13 Antimicrobial Performance

According to ISO 20743:2007, the absorption method was employed: Samples were inoculated with 1.0 × 10^5^ CFU/mL suspensions of *S. aureus* and *Escherichia coli* (*E. coli*), incubated at 37 °C for 24 h. Residual bacteria were eluted using a neutralizing solution, plated on agar, and incubated for colony counting (CFU). The antibacterial rate (AR) was calculated using the following formula

$$AR\;\left(\%\right)=\left(1-\frac{B}{A}\right)\times 100\%$$

Here, *B* is the CFU count for the treated sample, and *A* is the CFU count for the blank control.

### 2.14 In Vitro Clotting Properties

Specimens (8 mm × 8 mm × 2 mm) were prepared and placed in 50 mL centrifuge tubes. After incubation at 37 °C for 5 min, 200 μL of fresh anticoagulated blood was added to cover the sample surface, followed by 20 μL of CaCl_2_ solution to induce clotting. The samples were incubated in a 37 °C water bath.

At 5, 10, and 15 min, 50 mL of deionized water was added to dissolve uncoagulated blood cells. The samples were shaken at 50 rpm for 5 min and then left to stand. A portion of the supernatant was collected, and its absorbance at 545 nm was measured. Fresh anticoagulated blood diluted with an equal volume of deionized water served as the control (OD_C_, set at 100%). The blood clotting index (BCI) was calculated according to the below formula

$$BCI\;\left(\%\right)=\frac{{OD}_{S}}{{OD}_{C}}\times 100\%$$

Here, OD_S_ is the absorbance of the sample, and OD_C_ is the absorbance of the control.

### 2.15 Platelet Adhesion Properties

Lyophilized hydrogels (SA, SA-BBR@SFMP, and SA-BBR@SFMP-CGF) were prepared into 8 mm × 8 mm × 2 mm specimens, rinsed with PBS, and placed in a 24-well plate. Platelet-rich plasma (PRP) was obtained from fresh blood by centrifugation, and 100 μL of PRP was added dropwise onto the hydrogels. The samples were incubated at 37 °C on a shaker for 1 h.

The hydrogels were fixed with 2.5 wt % glutaraldehyde for 12 h, and then washed with PBS to remove loosely adhered platelets, followed by sequentially dehydration with ethanol solutions of 50%, 75%, 85%, 95%, and 100% (15 min each). Platelet adhesion on the hydrogel surfaces was observed *via* SEM.

### 2.16 Cell Culture and Seeding

To evaluate toxicity, L929 fibroblast cells were chosen according to FDA guidelines for intraarticular research devices.^21^ Lyophilized hydrogels (SA, SA-BBR@SFMP, and SA-BBR@SFMP-CGF) were cut to equal mass, sterilized with ultraviolet ozone, and extracted in serum-free DMEM (DMEM/PS = 100:1) at 5 mg/mL for 24 h. The extract was supplemented with 10% FBS and stored at 4 °C.

L929 cells were cultured and seeded at a density of 5000 cells per well in a 96-well plate. Experimental wells received 100 μL of hydrogel extract, while control wells contained 100 μL of complete medium. Each experimental group included six replicates, and the medium was replaced every 2–3 days. CCK-8 cell proliferation and live/dead staining assays were performed on days 1, 3, and 5.

### 2.17 Cell Viability Assay

A total of 110 μL CCK-8 solution (CCK-8:serum-free medium = 1:10) was added to each well and incubate in the dark for 2 h. Transfer 100 μL to a blank 96-well plate, using wells with 100 μL CCK-8 solution as the blank group. Measure absorbance at 450 nm with a microplate reader.^21^ Each sample includes six replicates, and cell viability (CV) is calculated using the following formula

$$CV\;\left(\%\right)=\frac{{OD}_{S}-{OD}_{B}}{{OD}_{C}-{OD}_{B}}\times 100\%$$

Here, OD_S_, OD_B_, and OD_C_ represent the OD values of the sample, blank, and control groups, respectively.

After 1, 3, and 5 days of culture, discard the medium and perform live/dead staining using Calcein-AM/PI. Add 100 μL of Calcein-AM/PI working solution (Calcein-AM/PI/buffer = 1:1:1000) to each well and incubate for 30 min. Triplicate samples were prepared for each time point and analyzed using a fluorescence inverted microscope.

### 2.18 Animal Model

Animal experiments were approved by the Ethics Committee of Soochow University and conducted in accordance with relevant regulations.^21^ Hydrogel dressings were swelled with PBS, punched into 10 mm diameter cylinders (approximately 2 mm height), sterilized *via* X-ray, and sealed for use.

Twelve male SD rats (60 g, 3 weeks old) were anesthetized with sodium pentobarbital (2 mL/kg) and given free access to food and water. After shaving and disinfecting the dorsal skin, a 10 mm circular full-thickness wound was created, followed by the application of 10 μL of *S. aureus* solution (10^6^ CFU/mL) to induce infection. Rats were divided into four groups (*n* = 5):

(1) Control group (no treatment).
(2) SA group.
(3) SA-BBR@SFMP group.
(4) SA-BBR@SFMP-CGF group.

Hydrogels were sterilized and applied to the wounds, covered with transparent sterile dressings and bandages (Figure S1). Dressings were changed regularly (Table S1), and wound photographs were taken to monitor healing. The wound healing ratio (WHR) was calculated using the below formula

$$WHR\;\left(\%\right)=\left(1-\frac{A_{t}}{A_{0}}\right)\times 100\%$$

Here, *A*_*t*_ and *A*_0_ represent wound areas on day *t* and day 0.

On days 7 and 14, rats were euthanized. Wound tissues were collected, fixed in 4% paraformaldehyde, and embedded in paraffin. Sections (5 μm thick) were stained with H&E, Masson, and CD31 for epidermal, vascular, and hair follicle regeneration. CK14 staining assessed skin thickness, and collagen deposition was evaluated. Results were imaged with a fluorescence microscope and analyzed using ImageJ.

### 2.19 Statistical Analysis

Each experiment was performed in triplicate, and results were expressed as mean ± standard deviation. Statistical analysis was conducted using SPSS 16.0. One-way ANOVA was applied to determine differences among multiple groups. A *p < 0.05 was considered statistically significant.

## 3 Results and Discussion

### 3.1 Demonstration of Composite Hydrogel with Drug-Release Capability and Enhanced Mechanical Performance

The SF solution is prepared through a multistep process, including degumming silk cocoons, extracting fibroin, and subsequent purification into an aqueous solution (Figure 1).^22^ The composite hydrogel dressing is prepared through two main steps (Figure 1b). SFMPs are produced *via* salting out leveraging self-assembly properties and biocompatibility of SF for drug encapsulation. These SFMPs are then combined with oppositely charged BBR to form BBR@SFMPs, while CGFs are treated with ethanol sonication to enhance their hydrophilicity by removing surface oil, thereby improving their dispersion in the composite mixture. The gradual release of Ca^2+^ through dialysis facilitates SA’s ionic cross-linking hydrogel formation. This design synergistically integrates the inherent biocompatibility and moisture-retentive properties of the SA hydrogel matrix, the sustained-release functionality of the BBR@SFMPs, and the mechanical reinforcement provided by CGFs. When applied to a rat wound model, the SA-BBR@SFMP-CGF exhibited superior wound healing performance. This effect is attributed to its potent antibacterial activity against *S. aureus* and its mechanical properties closely resembling those of native skin (Figure 1c).

**Figure 1:**
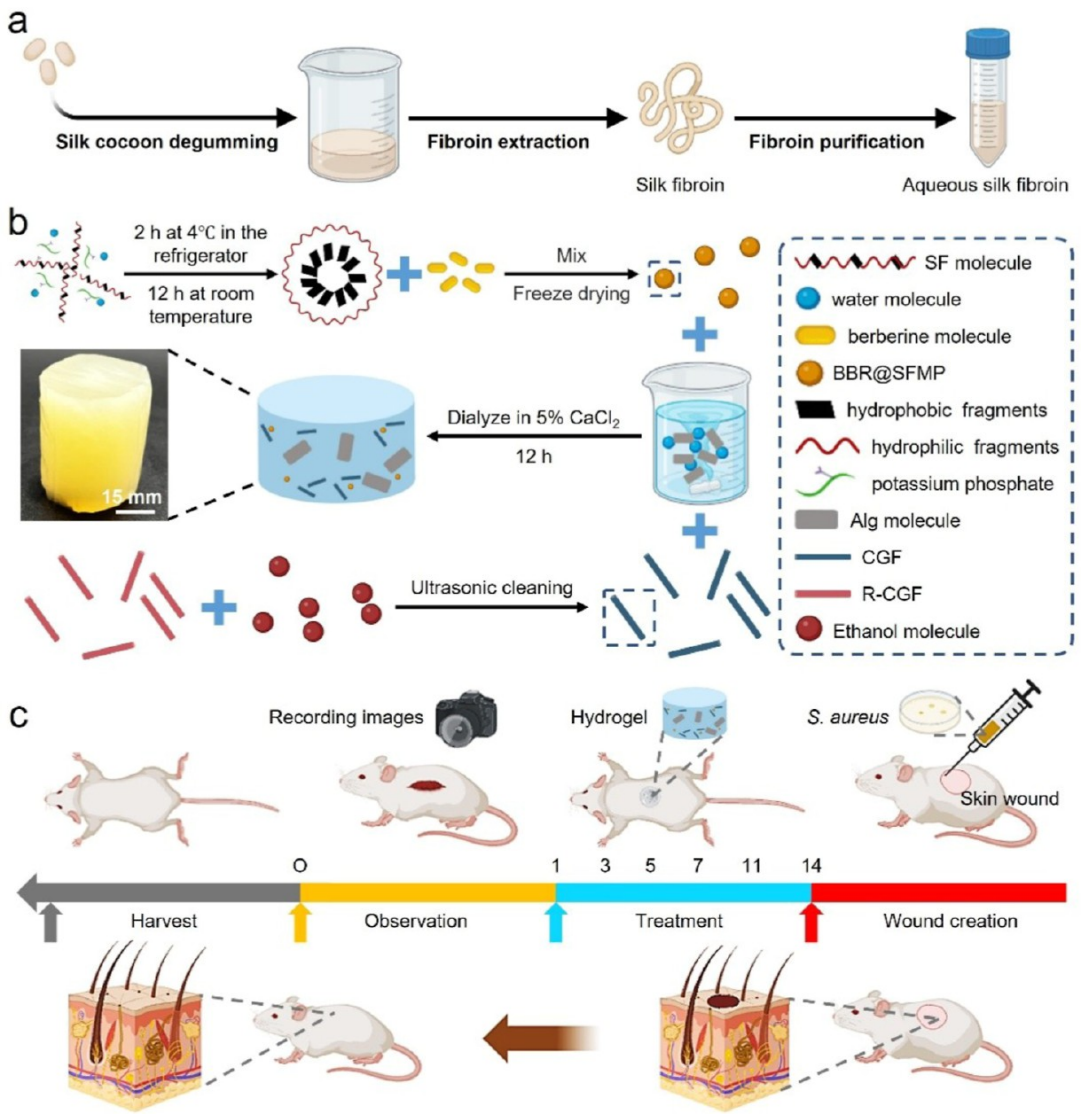
Preparation of composite hydrogels and their application in healing *S. aureus*-infected wounds. (a) Preparation process of SF solution; (b) preparation of SA-BBR@SFMP and SA-BBR@SFMP-CGF; (c) model and healing process of full-thickness skin wounds infected with *S. aureus*.

### 3.2 Microsphere Optimization

We explored the effect of SF concentration on the drug-loading and slow-release properties of microspheres as well as their optimal inhibitory concentration. SEM shows that 1% SFMP form uniform, smooth microspheres, while 5% and 10% SFMP exhibit increased roughness and heterogeneity, due to slower salting-out rates at higher SF concentrations (Figures 2a, S2 and Table S2). Excess SF concentrations limit microsphere formation by depleting phosphate availability, aligning with reported salting-out kinetics influencing microsphere uniformity and drug encapsulation efficiency.^17,23^ A linear regression equation of the absorbance over the drug concentration of BBR in deionized water was established using the scatter method (Figure S3a). The microspheres made with 1% SF have the highest yield (66.76 ± 5.34%) and drug-loading efficiency (29.76 ± 1.23%) (Figure S3b) (*p* < 0.01). It is attributed to sufficient phosphate for complete precipitation and the smaller particle size and larger specific surface area of 1% SFMP, which increases the likelihood of drug contact, leading to a higher drug loading capacity compared to 5% and 10% SFMP. Zeta potential measurements indicate that particle size and surface charge increase with SF concentration (Figure S3c). Following drug loading, the increased zeta potential confirms successful BBR incorporation. The larger zeta potential change in 10% SFMP suggests higher BBR surface adsorption. Increasing the BBR-to-SFMP ratio beyond 1:1 does not significantly improve the drug loading (*p* > 0.05) due to saturation of BBR (Figure S3d). Fourier transform infrared spectroscopy (FT-IR) identified characteristic SF secondary structures: random coil in SF at 1635–1648 cm^–1^, β-sheet at 1615–1635 cm^–1^, and α-helix appeared at 1650–1655 cm^–1^. β-sheet content increases with SF concentration, enhancing SFMP stability (Figure S4).^18^ After drug loading, 10% SFMP shows a significant β-sheet increase (*p* < 0.01) due to substantial surface drug loading. FTIR spectra show reduced absorption intensities of BBR characteristic peaks (2882 cm^–1^ and 1065 cm^–1^) postloading, confirming efficient encapsulation. Intact skin typically maintains an acidic pH (4–6), which shifts to physiological pH (7.4) during injury due to microvascular leakage, creating an environment conducive to bacterial infection.^24^ Release results reveal a diffusion-controlled mechanism (*n* < 0.45) at both pH 5.5 and 7.4 (Figure S5 and Table S3). Acidic conditions weaken hydrogen bonding and induce electrostatic repulsion, facilitating higher drug release, while neutral conditions restrict release due to stronger hydrogen bonding. BBR-loaded 1% SFMP (BBR@1%SFMP) achieves the most stable and prolonged release, while BBR-loaded 10% SFMP (BBR@10%SFMP) exhibits a higher initial release due to greater surface loading of BBR on the microspheres. Consequently, 1% SFMP with a 1:1 ratio is optimal for balancing high yield, drug loading and slow-release effects, and cost-effectiveness.

**Figure 2:**
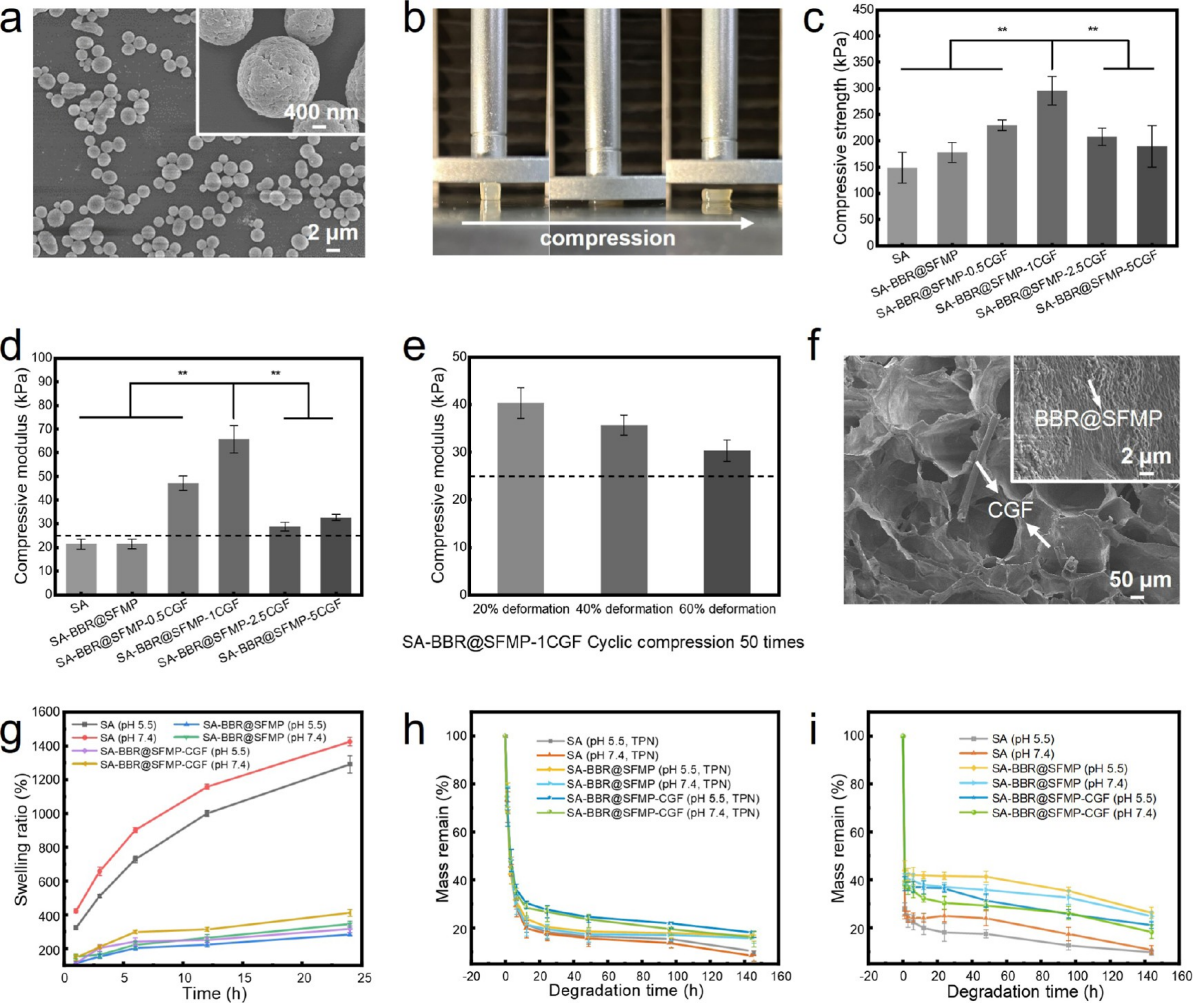
Characterization, mechanical properties, and degradation behavior of SA-BBR@SFMP-CGF composite hydrogels. (a) Surface morphology of BBR@1%SFMP after drug loading; (b) images of the compression process; (c) compression strength of the composite hydrogels; (d) compression modulus of the composite hydrogels; (e) compression modulus of SA-BBR@SFMP-CGF composite hydrogel dressings after 50 cycles at 20%, 40%, and 60% strain; (f) surface morphology of SA-BBR@SFMP-CGF; (g) swelling ratios of SA, SA-BBR@SFMP, and SA-BBR@SFMP-CGF at different pH values; (h) mass retention of SA, SA-BBR@SFMP, and SA-BBR@SFMP-CGF hydrogels in PBS and trypsin mixed degradation fluid; (i) mass retention of SA, SA-BBR@SFMP, and SA-BBR@SFMP-CGF hydrogels in PBS degradation fluid.

We explored the effect of BBR@1%SFMP concentration on the bacterial inhibitory properties. At the optimal inhibitory concentration (0.1 mg/mL), the inhibition against *S. aureus* is superior to that against *E. coli* (Figure S6). This difference is attributed to that *S. aureus* lacks an outer membrane compared to *E. coli*, consisting only of a single-layered cell membrane and a thick peptidoglycan layer, which allows BBR to penetrate more easily and exert its antimicrobial effects within the cell.

### 3.3 Mechanical Properties

Wound dressings for joints and other high-mobility areas must exhibit strong barrier protection and energy dissipation capabilities to ensure durability.^19^ We explored the effect of fiber incorporation on the compression properties of hydrogel. The addition of BBR@SFMPs increases the compressive stress and mechanical properties due to their uniform dispersion, which enhances its load-bearing capacity (Figure 2b–d and Table 2). With 3 mg of CGFs are incorporated, the SA-BBR@SFMP-CGF achieves a maximum compression strength 295.74 ± 27.45 kPa (Table S4). This enhancement arises from hydrogen bonding between the SA network and CGFs, as well as the uniform fiber distribution, which increases cross-linking density and facilitates energy dissipation. However, excess CGFs weaken the composite structure and reduce the compression strength because of the local fiber aggregation. An optimal amount of CGF improves mechanical strength and adjusts the compression modulus to match the Young’s modulus of human skin (25–600 kPa). In order to evaluate the cyclic stability of the hydrogel at a single selected strain level, the hydrogel is subjected to 50 consecutive compression cycles to simulate real-world usage (Figure S7). Initial strength loss occurs during the first 10 cycles, with larger strains causing greater reductions. However, after 20 cycles, both compression strength and modulus stabilize, and post-50 cycles, the modulus remains above 25 kPa (Figure 2e). Mechanical integrity under repeated stresses indicates that SA-BBR@SFMP-CGF can adapt effectively to body movements and provide continuous wound protection to promote healing.

**Table 2:** Experimental Design of Different BBR@SFMPs with CGFs Concentration

sample	BBR@SFMP concentration (mg/mL)	BBR@SFMP added to 40 mL water (mg)	CGF concentration (mg/mL)	CGF added to 40 mL water (mg)
SA	0	0	0	0
SA-BBR@SFMP	0.1	4	0	0
SA-BBR@SFMP-0.5CGF	0.1	4	0.025	1
SA-BBR@SFMP-1CGF	0.1	4	0.05	2
SA-BBR@SFMP-2.5CGF	0.1	4	0.125	5
SA-BBR@SFMP-5CGF	0.1	4	0.25	10

### 3.4 Morphology

Porosities of SA-BBR@SFMP-CGF exceed 80% (Figures 2f and S8, S9). Compared to SA hydrogels, the incorporation of BBR@SFMPs and CGFs reduces porosity due to increased hydrogen bonding and cross-linking density. Despite the reduced porosity, the hollow structure of CGFs still supports cell proliferation, migration, and adhesion. The porous nature of the hydrogel allows it to absorb wound exudate effectively and facilitate the exchange of oxygen, water, and nutrients, which are critical for cellular activity and extracellular matrix deposition. This ensures an environment conducive to wound healing, particularly by promoting cell adhesion and migration at the wound site.^20,25^

### 3.5 Swelling Performance

Within 24 h, SA hydrogel continues to expand, demonstrating the superior water absorption and swelling properties (Figures 2g and S10a). The swelling ratios of SA-BBR@SFMP and SA-BBR@SFMP-CGF are significantly lower than that of the SA hydrogel (**p < 0.01) (Table S5). This reduction is attributed to the increased cross-linking density and decreased porosity from the incorporation of BBR@SFMPs and CGFs, as compared to the SA hydrogel (**p < 0.01). SA-BBR@SFMP-CGF shows a higher swelling ratio than SA-BBR@SFMP because CGFs can absorb more wound exudate due to their hollow structure. Furthermore, the hydrogels absorb more water at pH 7.4 compared to pH 5.5, where swelling decreases due to electrostatic attraction between ions that contracts the network. This pH-dependent behavior supports the effective absorption of exudate by the hydrogel during the initial wounding phase (pH 7.2–8.0) and ensures the effective release of BBR to inhibit bacteria, promoting effective healing.^20^ The swelling behavior of hydrogel dressings not only reflects their water absorption capacity but also influences their degradation performance to some extent.

### 3.6 In Vitro Degradation Performance

All hydrogels degrade rapidly within the first 24 h (Figures 2h,i and S10b,c). After this phase, degradation slows, especially in pH 5.5 environments. The degradation rate of SA-BBR@SFMP-CGF is slower to support sustained antibacterial effects due to the inclusion of SFMPs and CGFs. This degradation pattern allows rapid inhibition of bacterial growth in the early stages and sustained inhibition in the later stages. Moreover, the degradation products meet the requirements of nontoxicity.^21^

### 3.7 Drug Release Performance and In Vitro Antimicrobial Performance

The release of BBR from SA-BBR@SFMP-CGF achieves an effective concentration quickly, with a release rate of 18.80 ± 0.82% within 1 h at pH 7.4, surpassing the required 15.87%. Drug release occurs more rapidly at pH 7.4 than at pH 5.5 due to the network expansion at the higher pH. Drug release from SA-BBR@SFMP and SA-BBR@SFMP-CGF is influenced by the degree of hydrogel cross-linking, with SA-BBR@SFMP-CGF showing slower release due to stronger hydrogen bonding between CGFs and SFMPs. Furthermore, the SF microspheres themselves provide a robust platform for sustained release, as the β-sheet structure of SF stabilizes the microspheres and modulates drug diffusion, complementing the barrier effect imposed by the SA hydrogel network and CGF interactions. The skin serves as the body’s protective barrier and the first line of defense against bacterial infections.^26^ Incorporating BBR@SFMPs into composite hydrogel dressings provides long-lasting antimicrobial protection to prevent infections and support wound healing. In SA-BBR@SFMP-CGF, BBR is loaded onto SFMP, resulting in a slower release rate. The BBR release rate drops from over 30% to less than 20% within the first hour and from full release to under 40% at the 120th hour, compared to direct loading of BBR into SA hydrogels.

We investigated the antimicrobial properties of SA-BBR@SFMP-CGF. After 24 h of bacterial culture, the growth values for *E. coli* (2.53 ± 0.13) and *S. aureus* (2.22 ± 0.24) are both greater than 0.7. BBR@SFMPs provide the antimicrobial effect, especially against *S. aureus*, which shows stronger inhibition compared to *E. coli* (**p < 0.01) (Figure 3a–c and Table S6). SA-CGF hydrogels exhibit no antibacterial activity, confirming that the antimicrobial properties are mainly attributed to BBR.

**Figure 3:**
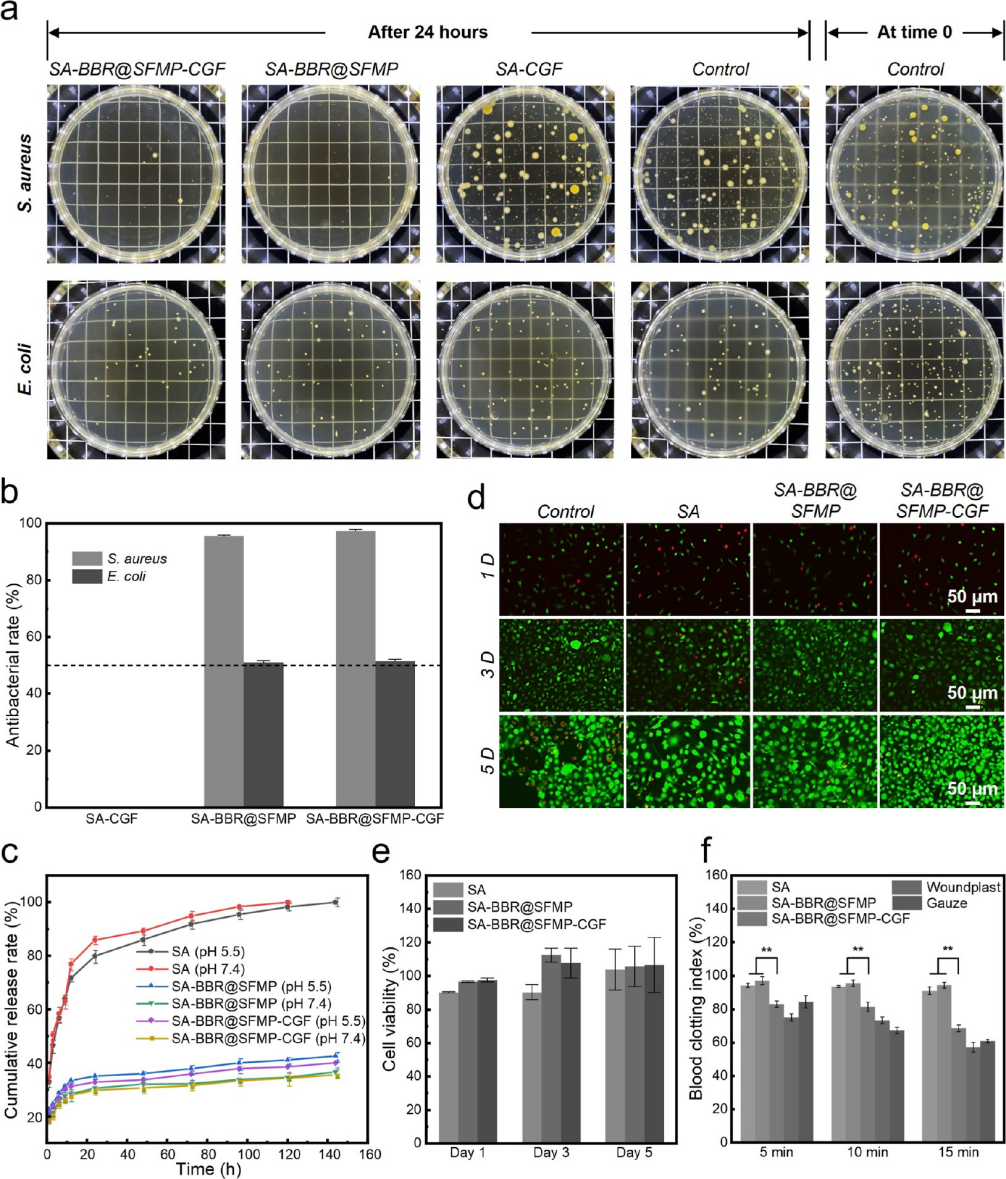
Antimicrobial activity, drug release, and biocompatibility of SA-BBR@SFMP-CGF composite hydrogels. (a) Antimicrobial characterization of SA-CGF, SA-BBR@SFMP, and SA-BBR@SFMP-CGF against *S. aureus* and *E. coli*; (b) antimicrobial performance of SA-CGF, SA-BBR@SFMP, and SA-BBR@SFMP-CGF against *S. aureus* and *E. coli* (50%–90% indicates bacteriostatic effect, >90% indicates strong bacteriostatic effect); (c) the drug release profiles of SA, SA-BBR@SFMP, and SA-BBR@SFMP-CGF in two types of PBS buffer solutions; (d) fluorescent staining results of mouse fibroblast cells cultured in the extract of different composite hydrogel dressings; (e) statistical chart of cell proliferation results using CCK-8 assay for composite hydrogel dressings; (f) statistical chart of blood clotting index.

### 3.8 In Vitro Cytocompatibility

The excellent biocompatibility of both SA and SF, key components of the composite hydrogels, was reflected in the cell culture results. After 1 day, cells in all experimental groups show good viability, exhibiting polygonal or spindle-shaped morphologies. While the live cell density is slightly higher in the control group, the number of dead cells remains consistent across all groups. By day 3, live cells predominantly exhibit spindle-shaped morphology and have filled the culture dish, with the SA-BBR@SFMP group showing the highest cell density, followed by SA-BBR@SFMP-CGF, both displaying fewer dead cells. By day 5, live cells are more densely packed, with some areas showing cell depletion, and the cells have transitioned from spindle-shaped to round, indicating reduced viability (Figures 3d and S11). This shift is attributed to glycoproteins on the cell membrane, which limits further proliferation once a certain cell density is reached. Experimental hydrogel groups outperform the control group in maintaining cell viability by providing additional nutrients. Cell viability remains above 90% in all groups after 1, 3, and 5 days (Figure 3e). SA-BBR@SFMP-CGF achieves 106.71 ± 16.39% viability on day 5, demonstrating excellent biocompatibility. The SA-BBR@SFMP and SA-BBR@SFMP-CGF groups show superior cell proliferation compared to the control group, attributed to the presence of SF. The hollow structure of CGFs enhances conditions for cell migration and proliferation without compromising compatibility.

### 3.9 Hemocompatibility and Hemostatic Performance

The positive control solution appears blood-red due to hemolysis, releasing hemoglobin from ruptured red blood cells. In contrast, solutions from the negative control and hydrogel groups remain clear, with red blood cells intact and no hemolysis (Figure S12a). With a hemolysis rate below 5%, all three hydrogel groups indicate excellent hemocompatibility (Figure S12b).^27,28^ The incorporation of CGFs into SA-BBR@SFMP-CGF improves its hemostatic properties, as demonstrated by its lower BCI. In 5 min, the BCI of SA-BBR@SFMP-CGF is significantly lower (**p < 0.01) than that of SA and SA-BBR@SFMP, outperforming commercial gauze in hemostatic performance. Over time (10–15 min), its effectiveness becomes comparable to two commercial hemostatic products (Figures 3f and S13). In platelet adhesion results, SA-BBR@SFMP-CGF shows the densest platelet aggregation, while SA-BBR@SFMP exhibits less aggregation. Platelet aggregation depends on fibrin network formation,^29^ and due to the increased surface electronegativity of BBR@SFMP, the monomers are less likely to polymerize into fibrin polymers to form a stable network, thus reducing platelet aggregation. With the addition of CGF, its hollow structure provides physical support for the deposition of platelets and fibrin and improves coagulation (Figure S14). This synergistic interaction makes SA-BBR@SFMP-CGF particularly effective in rapid hemostasis. These results suggest that the materials are blood-friendly and may enhance their clinical applications.

### 3.10 In Vivo Biocompatibility of Composite Hydrogel

By day 3 postoperation, the control group shows signs of *S. aureus*-induced inflammation, including red blood, purulent exudate. By day 5, black necrotic areas become evident due to tissue congestion, necrosis, and inflammatory infiltration. These necrotic areas persist, although reduced in size, until day 14, indicating slow wound healing (Figure 4a–d). In contrast, the SA, SA-BBR@SFMP, and SA-BBR@SFMP-CGF hydrogel groups exhibit significantly accelerated healing from day 5 onward (**p < 0.01), with complete wound closure by day 14, particularly in the SA-BBR@SFMP-CGF group, which outperforms the other groups in both healing speed and quality (Figure 4e,f). Despite the delayed healing caused by *S. aureus* infection, these hydrogels demonstrate excellent wound healing properties. The SA-BBR@SFMP and SA-BBR@SFMP-CGF groups achieve superior healing outcomes.

**Figure 4:**
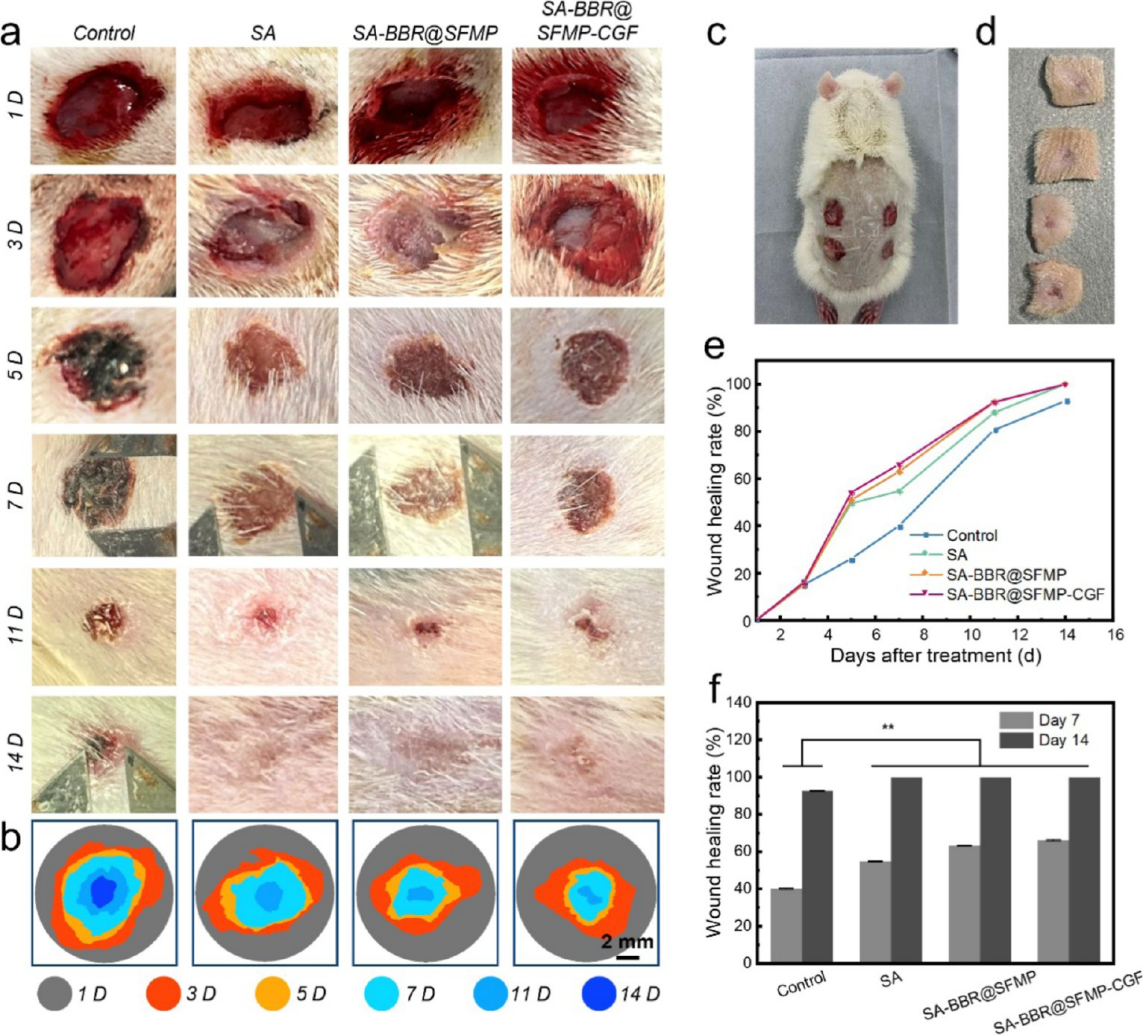
Evaluation of wound healing efficacy of SA-BBR@SFMP-CGF hydrogels in SD rats. (a) Photographs depicting the healing progress of SD rat dorsal skin wounds treated with Control, SA, SA-BBR@SFMP, and SA-BBR@SFMP-CGF hydrogels on day 1, 3, 5, 7, 11, and 14; (b) schematic representation of wound healing processes in SD rats representing the control group and different samples; (c) coverage of wounds with hydrogel; (d) partially healed skin images on day 7 (From top to bottom, SA-BBR@SFMP-CGF, SA-BBR@SFMP, SA and Control, the control group did not do any treatment, and the other groups covered the wounds with thoroughly sterilized hydrogel respectively); (e) healing progress of wounds after treatment with different samples; (f) statistical table of wound healing rates on day 7 and 14 after treatment with different samples. *n* = 5. *p < 0.05.

On day 7, the control group shows incomplete epithelial regeneration with tissue necrosis, while the SA, SA-BBR@SFMP, and SA-BBR@SFMP-CGF groups exhibit signs of epithelial regeneration. Among them, the SA-BBR@SFMP-CGF group indicates superior tissue repair, with the smoothest and most uniform regeneration, showing no significant necrosis or abnormal granulation tissue (Figure 5a). The presence of new blood vessels and hair follicles accelerate tissue repair.^30,31^ The control group demonstrates minimal neovascularization and few new hair follicles, while the SA-BBR@SFMP-CGF group significantly enhances both (**p < 0.01) (Figure 5b). By day 14, neovascularization further increases. The control group still exhibits incomplete and disordered epithelial regeneration with the fewest new blood vessels and hair follicles. In contrast, the SA-BBR@SFMP and SA-BBR@SFMP-CGF groups demonstrate effective treatment for infected wounds, as evidenced by the significant neovascularization and formation of new hair follicles, along with well-organized skin tissue (Figure 5c). Overall, the SA-BBR@SFMP-CGF group achieves the best healing outcomes, attributed to multiple factors: the SA hydrogel matrix reliably maintains a moist wound environment conducive to cell migration and proliferation; the SF component, both in the microspheres and likely contributing to the matrix biocompatibility, supports cellular activities; and BBR released from the SF microspheres effectively inhibits the growth and infection of *S. aureus*. This combined action reduces inflammation and further accelerates wound healing.

**Figure 5:**
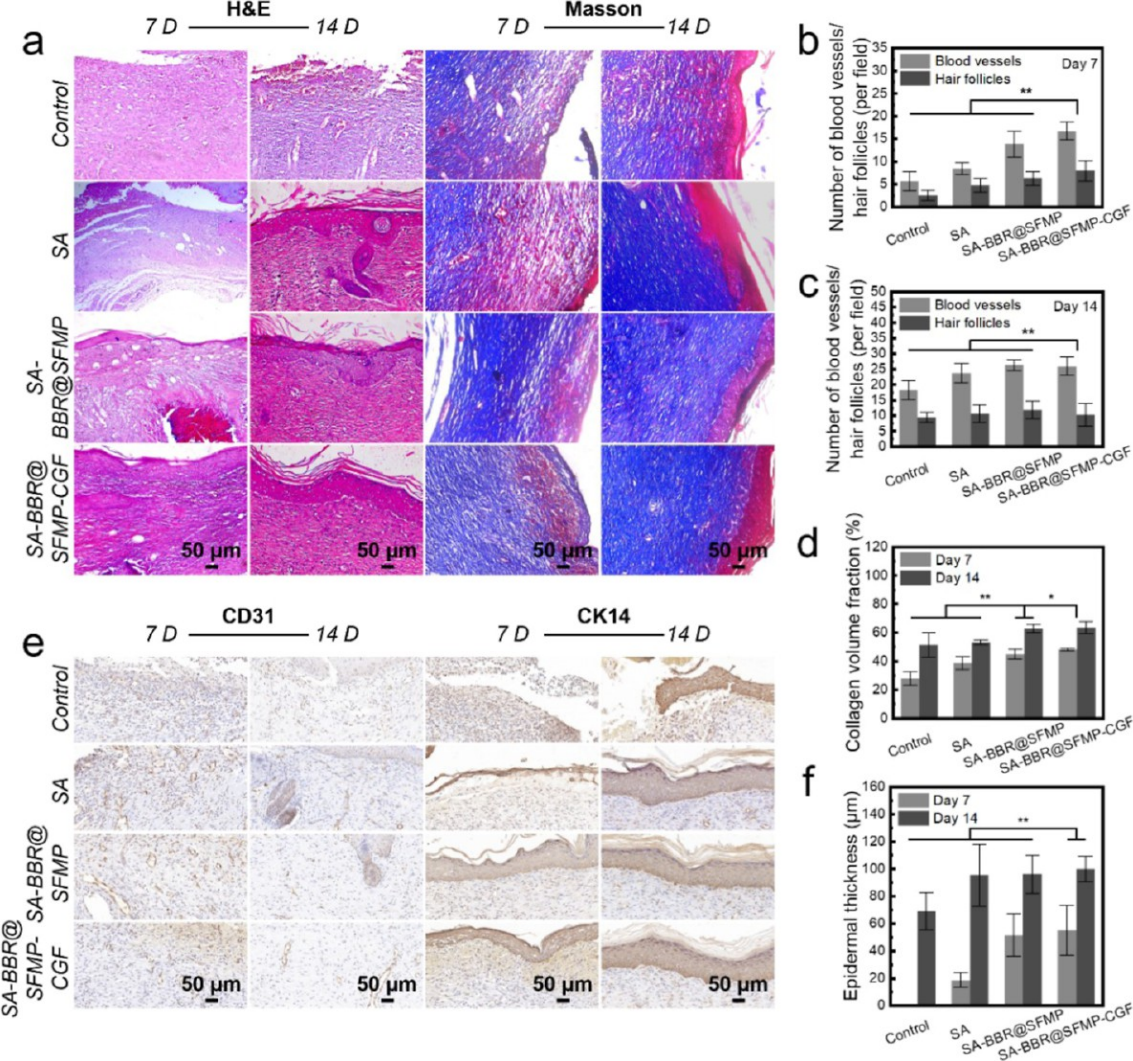
Histological analysis and tissue regeneration of wounds treated with SA-BBR@SFMP-CGF hydrogels. (a) H&E and Masson’s trichrome staining results of tissue from SD rat dorsal skin wounds treated with Control, SA, SA-BBR@SFMP, and SA-BBR@SFMP-CGF groups on day 7 and 14; (b,c) statistical table of neovascularization and new hair follicles on day 7 and 14 after treatment with different samples; (d) statistical table of collagen deposition rates on day 7 and 14 after treatment with different samples of wounds; (e) CD31 and CK14 staining results; (f) statistical table of epidermal thickness on day 7 and 14 after treatment with different samples of wounds.

Collagen is essential for maintaining tissue structure and function, retaining skin moisture, preventing dryness, and stimulating cell regeneration, all of which promote wound healing.^32–36^ Among the groups, the SA-BBR@SFMP-CGF group demonstrates the most pronounced regenerative epidermis, with more orderly subcutaneous tissue and denser collagen deposition. On days 7 and 14, collagen deposition in the SA-BBR@SFMP-CGF group is significantly higher than in the control and SA groups (**p < 0.01) and surpasses the SA-BBR@SFMP group (*p < 0.05), indicating its superior wound healing ability (Figure 5a–d).

CK14 staining reveals more pronounced epidermal regeneration in the SA-BBR@SFMP-CGF group. By day 14, all groups show a significant increase in epidermal thickness (*p < 0.05), with the SA-BBR@SFMP-CGF group achieving the most notable improvement. These findings highlight the exceptional potential of SA-BBR@SFMP-CGF in enhancing skin proliferation, repair, and overall wound healing (Figure 5e,f).

## 4 Conclusions

In summary, we have developed a novel SA-BBR@SFMP-CGF composite hydrogel dressing using CGF, SF, and SA as base materials, fabricated *via* salting-out and freeze-drying techniques. In contrast to traditional dressings with limited drug release performance, our hydrogel dressing incorporates BBR@SFMPs prepared *via* a two-step method, achieving sustained and controlled drug release that conforms to the Ritger-Peppas model. This design significantly enhances drug bioavailability and provides long-lasting antibacterial effects, particularly against *S. aureus*. Additionally, to address the mechanical limitations of traditional hydrogel dressings, we innovatively introduced CGF as a reinforcing component. This integration substantially improves the compressive strength and flexibility of the hydrogel dressing. The incorporation of hollow-structured CGFs contributes to improved hemostatic properties and enables a slower drug release, allowing the dressing to better adapt to the complex wound shapes and accommodate dynamic movements. These advancements are of great significance as the synergistic effects of drug release and mechanical enhancement not only extend the dressing’s usage duration and reduce replacement frequency but also offer prolonged wound protection, improve patient comfort, and accelerate wound healing. We anticipate that the strategy of integrating drug release and mechanical reinforcement will demonstrate substantial value in wound treatment, infection control, and chronic disease care.

## Supplementary Material



## Notes

Wound infection healing experiments in mice, surface morphology of SFMP and BBR@SFMP, linear regression equations for BBR concentration in deionized water, characterization of BBR drug loading and yield, characterization of zeta potential and particle size, characterization of the ratio of BBR to SFMP mass of the input drug, FT-IR characterization and changes in secondary structure of SFMP and BBR@SFMP, linear regression equation of absorbance vs drug concentration, *in vitro* drug release profiles, bacterial inhibition rate, compression stress–strain curves of SA-BBR@SFMP-1CGF composite hydrogel dressings, surface morphology, pore size distribution and dissolution testing and mass remain of SA, SA-BBR@SFMP and SA-BBR@SFMP-CGF hydrogels, fluorescence staining images, hemolysis experiments, coagulation index test experiment, SEM images of platelet adhesion properties, yield, particle size, potential, loading of different samples rate, *in vitro* release kinetic modeling of drug-carrying microspheres in different pH, compression strength of composite hydrogels, swelling ratios of hydrogel dressings at different pH and antimicrobial performance of composite hydrogel dressings (PDF)
