# Supplementary material for: Composite Hydrogel Dressing with Drug-Release Capability and Enhanced Mechanical Performance
Source: Biomacromolecules. 2025 Aug 12;26(9):5715–26. doi: 10.1021/acs.biomac.5c00505 (PMC12421659; doi:10.1021/acs.biomac.5c00505)
Supplement: Supplementary file 1 [file bm5c00505_si_001.pdf]

Supporting Information

Composite Hydrogel Dressing with Drug-Release Capability and Enhanced Mechanical Performance

Jie Yang [a, #], Fanlei Yang [b, #], Wenhao Xu [a, #], Xiuqin Yu [c], Zhaozhu Zheng [a], Xiaoqin Wang [a], Kaili Chen [d, e, *], Jia Yu [f, *], Gang Li [a, *]

[a] National Engineering Laboratory for Modern Silk, College of Textile and Clothing Engineering, Soochow University, Suzhou 215123, China.

[b] Orthopedic Institute, Department of Orthopaedic Surgery, The First Affiliated Hospital, Suzhou Medical College, Soochow University, Suzhou 215006, China.

[c] Department of Dermatology, the First Affiliated Hospital of Soochow University, Suzhou 215006, China

[d] Department of Materials, Imperial College London, Exhibition Road, London SW7 2AZ, UK.

[e] Botnar Research Center, University of Oxford, Old Road, Headington, Oxford OX3 7LD, UK.

[f] School of Physical Education and Sports, Soochow University, Suzhou, 215006, China

[#]These authors contributed equally to this work.

[*]Correspondence and requests for materials should be addressed to

Kaili Chen

Department of Materials, Imperial College London, Exhibition Road, London SW7 2AZ, UK; Botnar Research Center, University of Oxford, Old Road, Headington, Oxford OX3 7LD, UK.

Email address: kaili.chen@ndorms.ox.ac.uk (Kaili Chen)

Jia Yu

School of Physical Education and Sports, Soochow University, Suzhou, 215006, China.

Email address: jiayu@suda.edu.cn (Jia Yu)

Gang Li

National Engineering Laboratory for Modern Silk, College of Textile and Clothing Engineering, Soochow University, Suzhou 215123, China.

Email address: tcligang@suda.edu.cn (Gang Li)

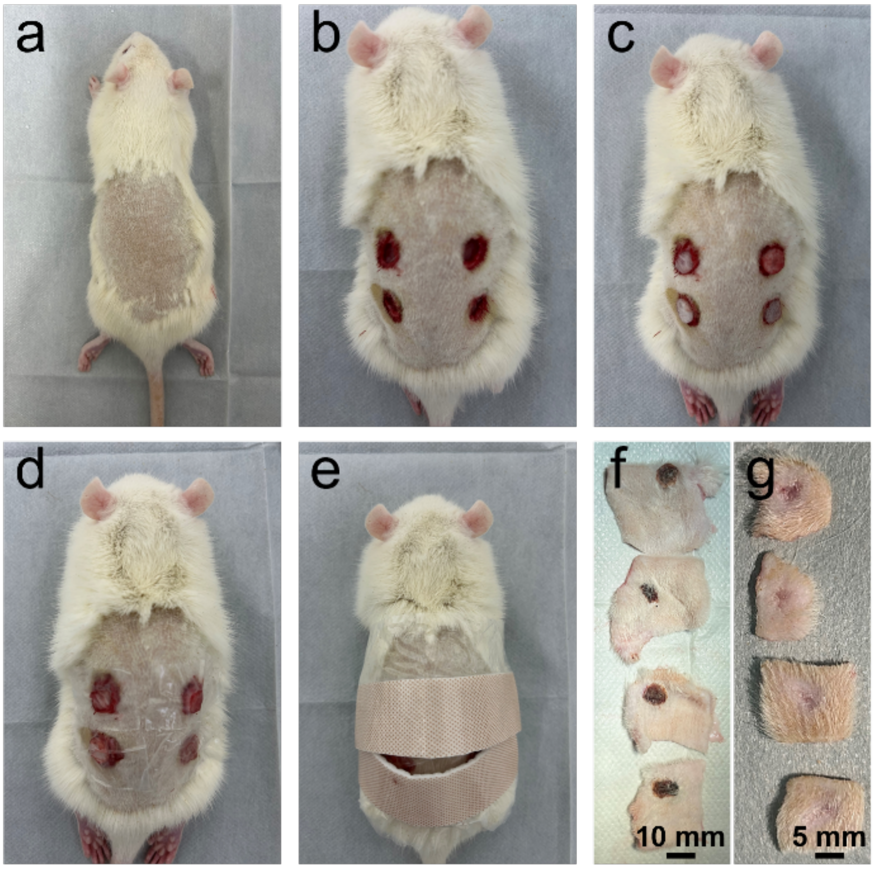

Figure S1. Wound infection healing experiments in mice. (a) Establishment of shaved and infected wound model; (b) 10 μL of *S. aureus* bacterial solution (10^6 CFU/mL) was dropped onto a circular, full-length skin wound with a diameter of 10 mm to infect the wound; (c) Cover the wound with a thoroughly sterilized hydrogel; (d) Cover with a clear sterile patch for wound visualization; (e) Dressing to cover the wound and hold the hydrogel in place; (f-g) Partially healed skin images on day 7 and day 14.

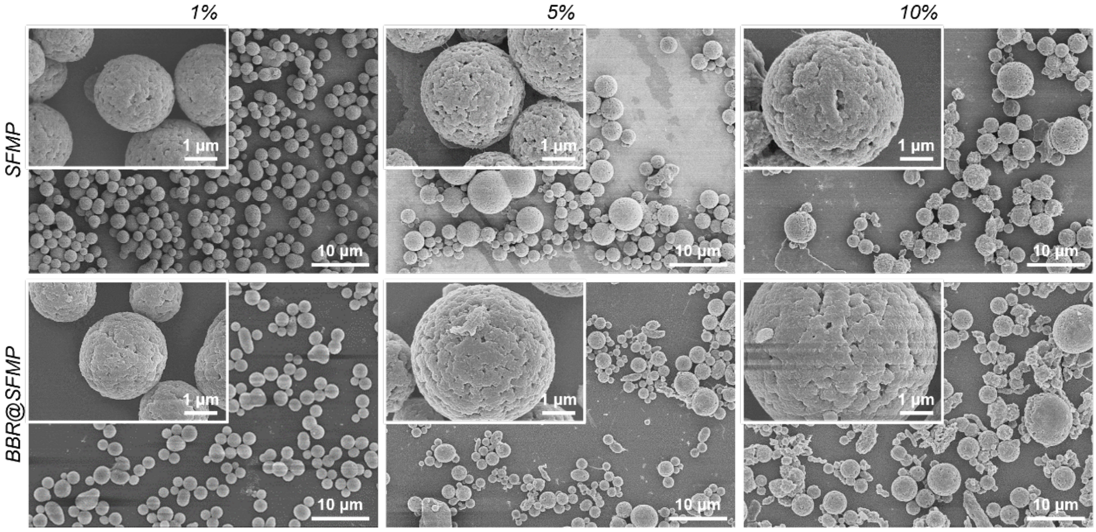

Figure S2. Surface morphology of SFMP and BBR@SFMP made by 1%, 5%, 10% mass fraction.

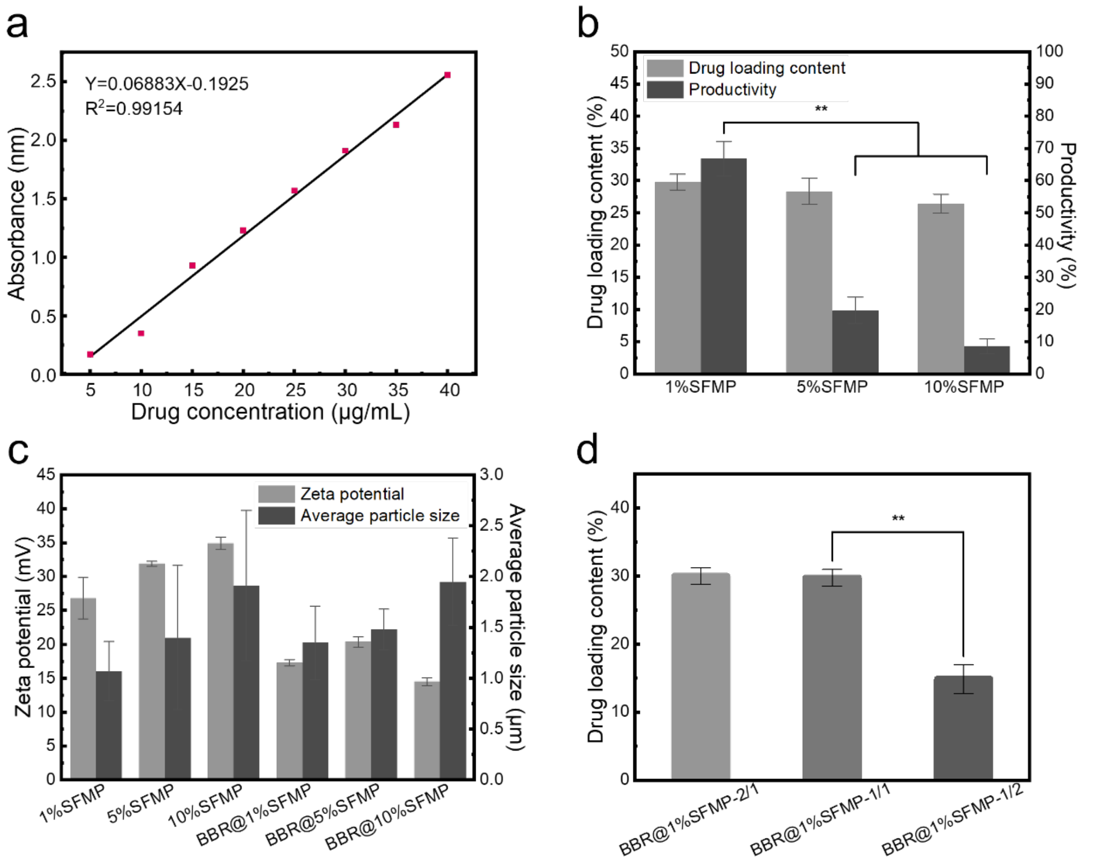

Figure S3. Drug-carrying properties of different concentrations of SFMP and related test characterization. (a) Linear regression equations for BBR concentration in deionized water; (b) Characterization of BBR drug loading and yield by 1%, 5%, and 10% mass fraction of SFMP; (c) Characterization of zeta potential and particle size of 1%, 5%, and 10% mass fraction of SFMP vs. BBR@SFMP; (d) Characterization of the ratio of BBR to SFMP mass of the input drug (2/1, 1/1, 1/2).

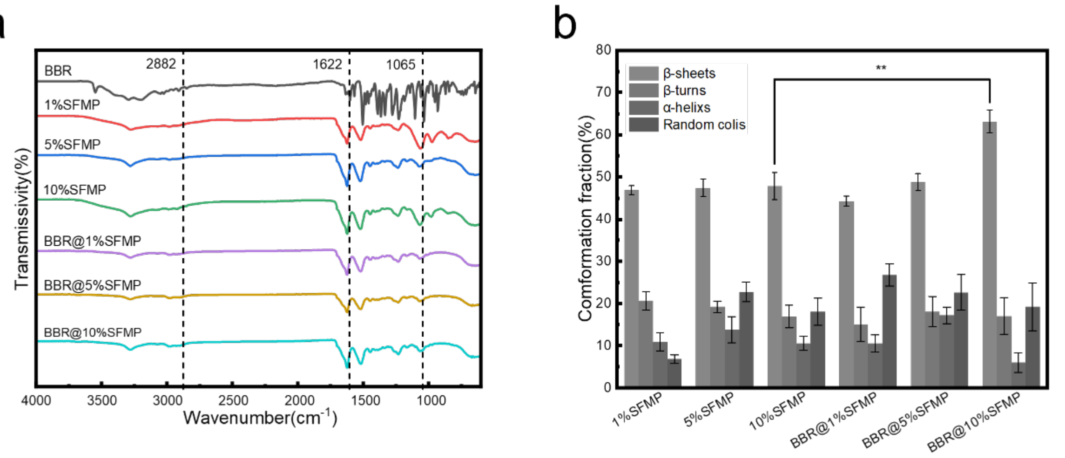

Figure S4. Characterization of different concentrations of SFMP before and after drug loading correlation tests. (a) FT-IR characterization of SFMP vs. BBR@SFMP at 1%, 5%, and 10% mass fractions; (b) Changes in secondary structure of SFMP and BBR@SFMP at 1%, 5%, and 10% mass fractions.

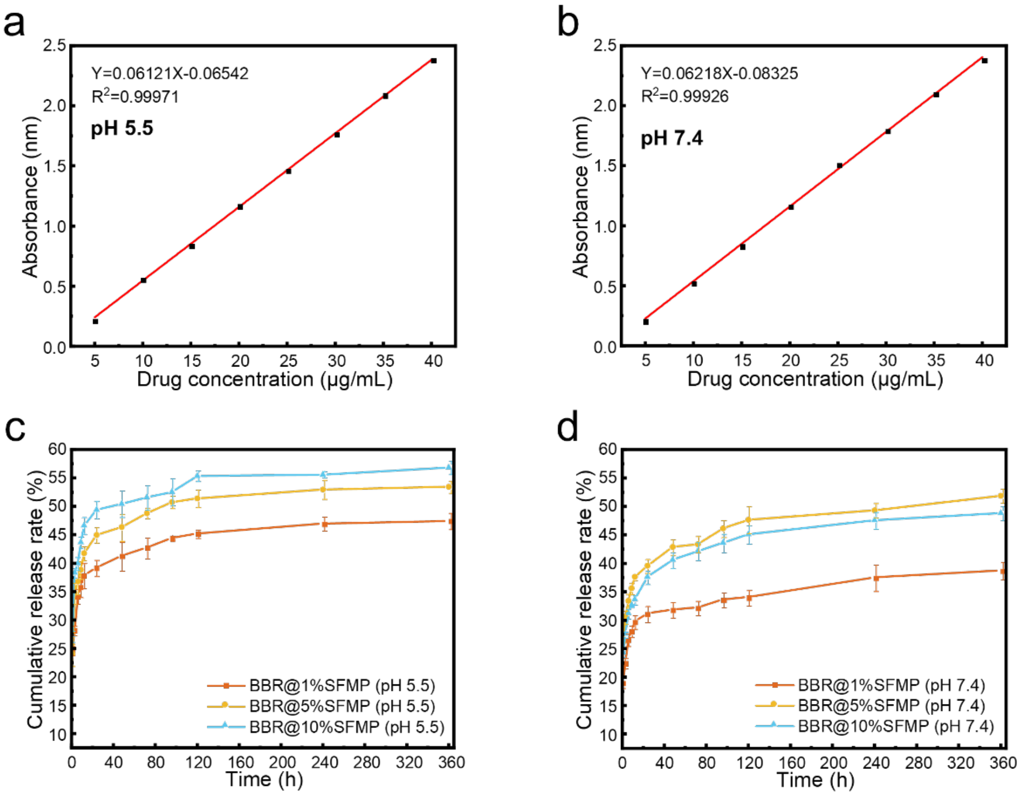

Figure S5. Linear regression equation of absorbance vs. drug concentration in BBR dissolved in PBS solution and *in vitro* drug release profiles of 1%, 5% and 10% SF-loaded antimicrobial slow-release microspheres. (a-b) Linear regression equations of absorbance vs. drug concentration of BBR dissolved in 10 mmol/L PBS at pH 5.5 and pH 7.4 solutions, respectively; (c-d) *In vitro* drug release profiles of 1%, 5% and 10% drug-loaded antimicrobial slow-release microspheres at pH 5.5 and pH 7.4.

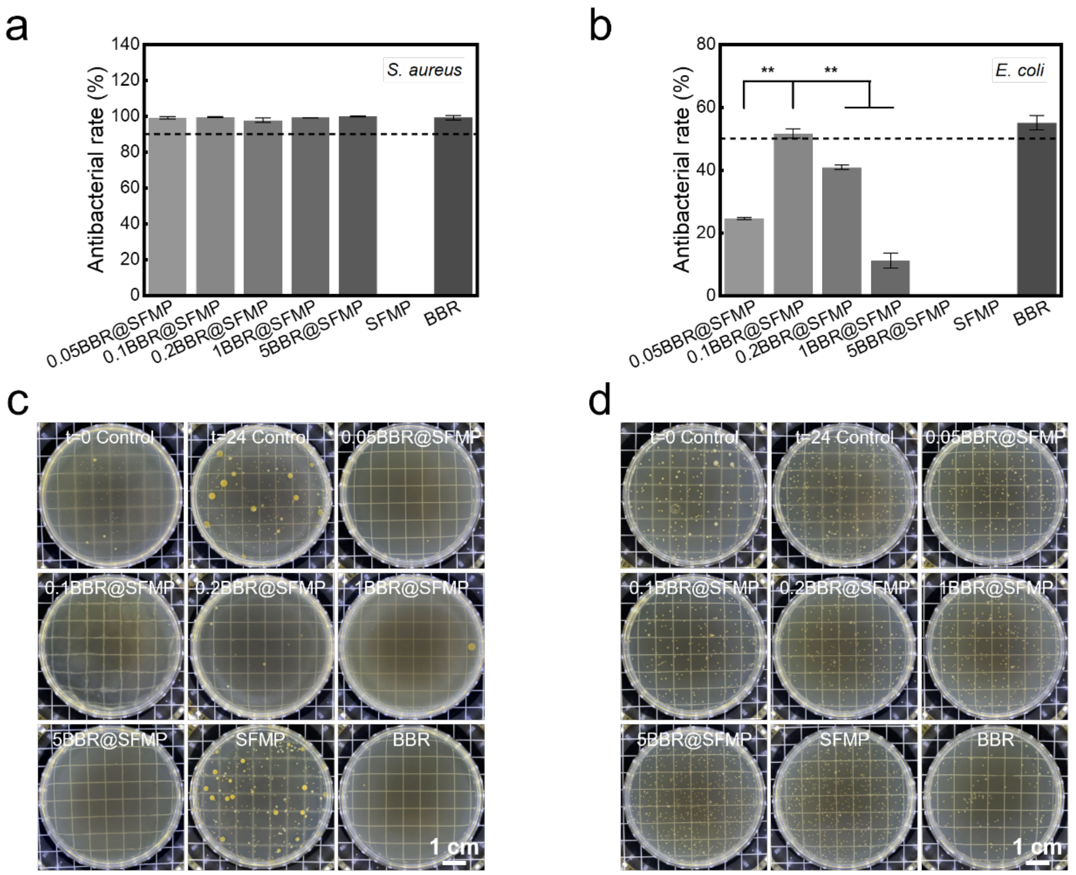

Figure S6. Bacterial inhibition rate of different concentrations of drug-loaded antibacterial slow-release microspheres, SFMPs and BBR plotted against bacterial experiments. (a-b) Antibacterial rate of the samples against *S. aureus* and *E. coli*; (c-d) Control samples at t_0 before incubation, bacterial growth of Control samples at t_0 before incubation, 0.05, 0.1, 0.2, 1, 5BBR@1% SFMP, SFMP and BBR after 24-h incubation with *S. aureus* (c) and *E. coli* (d).

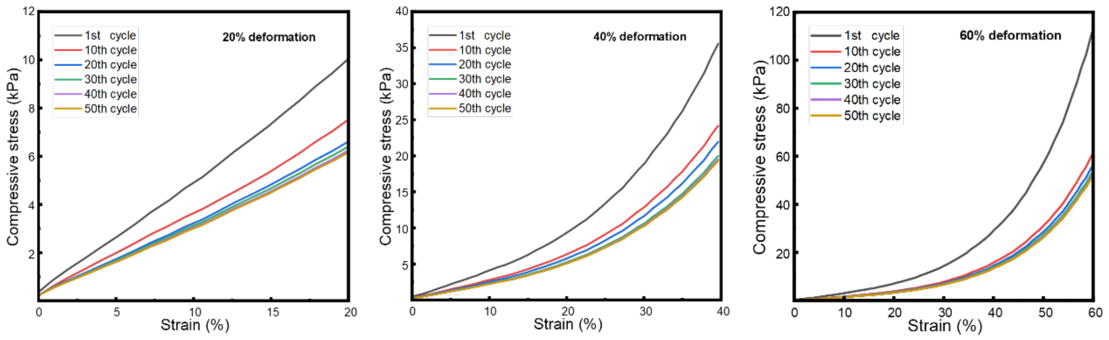

Figure S7. Compression stress-strain curves of SA-BBR@SFMP-1CGF composite hydrogel dressings at 20%, 40%, and 60% strain for the 1st, 10th, 20th, 30th, 40th, and 50th cycles.

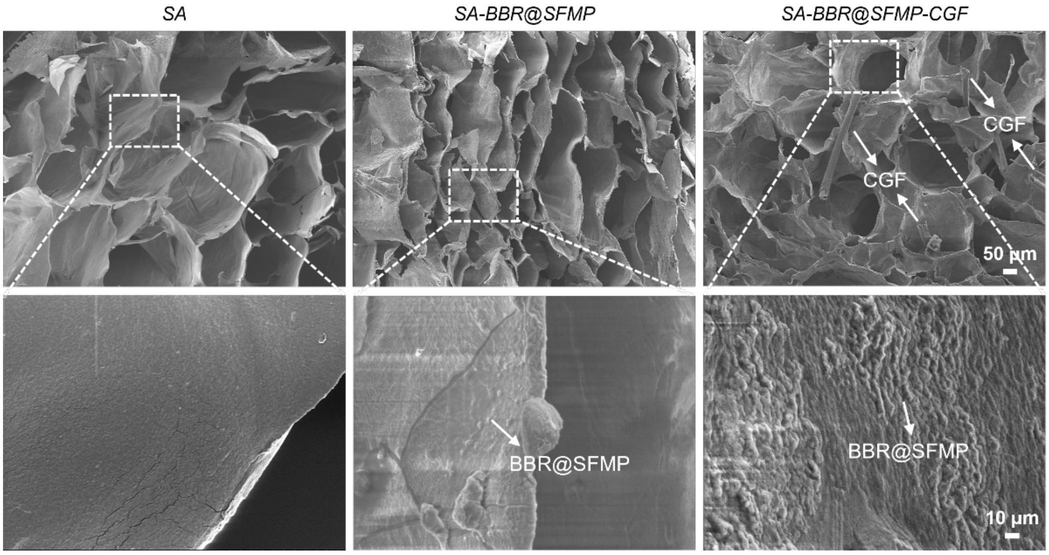

Figure S8. Surface morphology of SA, SA-BBR@SFMP and SA-BBR@SFMP-CGF hydrogels.

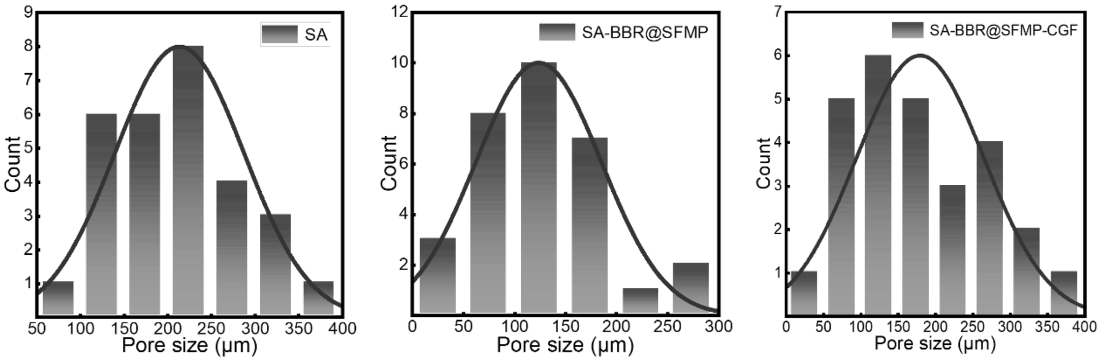

Figure S9. Pore size distribution of SA, SA-BBR@SFMP and SA-BBR@SFMP-CGF.

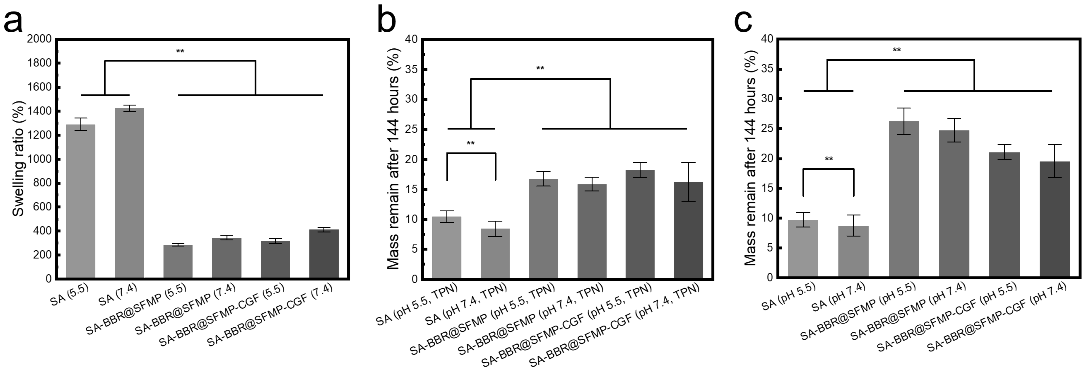

Figure S10. Dissolution testing and mass remain after 144 h of different samples. (a) Dissolution rates of SA, SA-BBR@SFMP, and SA-BBR@SFMP-CGF at different pH values; (b) Mass remain after 144 h of SA, SA-BBR@SFMP, and SA-BBR@SFMP-CGF hydrogels in PBS and trypsin mixed degradation fluid; (c) Mass remain after 144 h of SA, SA-BBR@SFMP and SA-BBR@SFMP-CGF hydrogels in PBS degradation fluid.

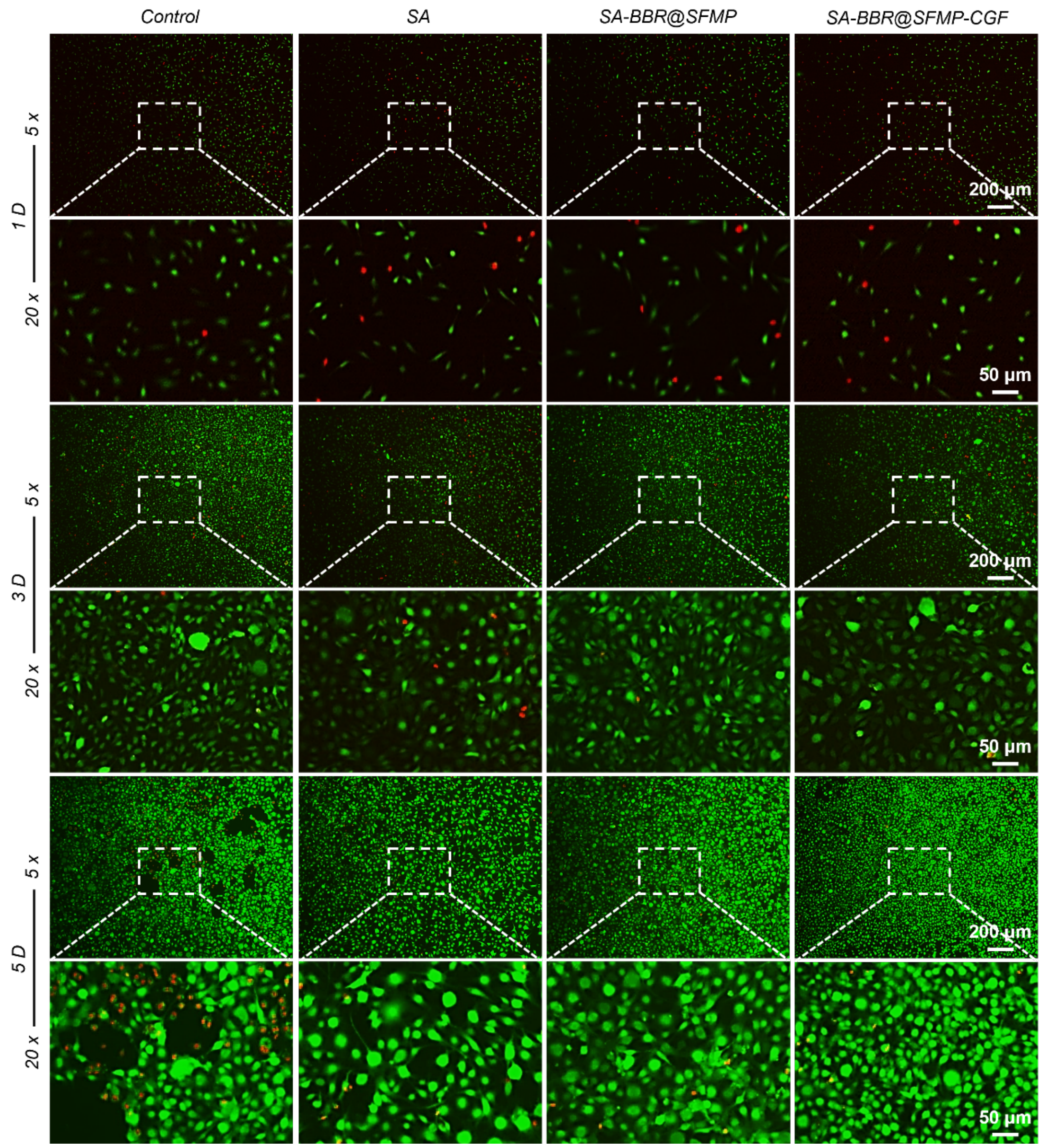

Figure S11. Fluorescence staining images of Control, SA, SA-BBR@SFMP and SA-BBR@SFMP-CGF on day 1, 3 and 5.

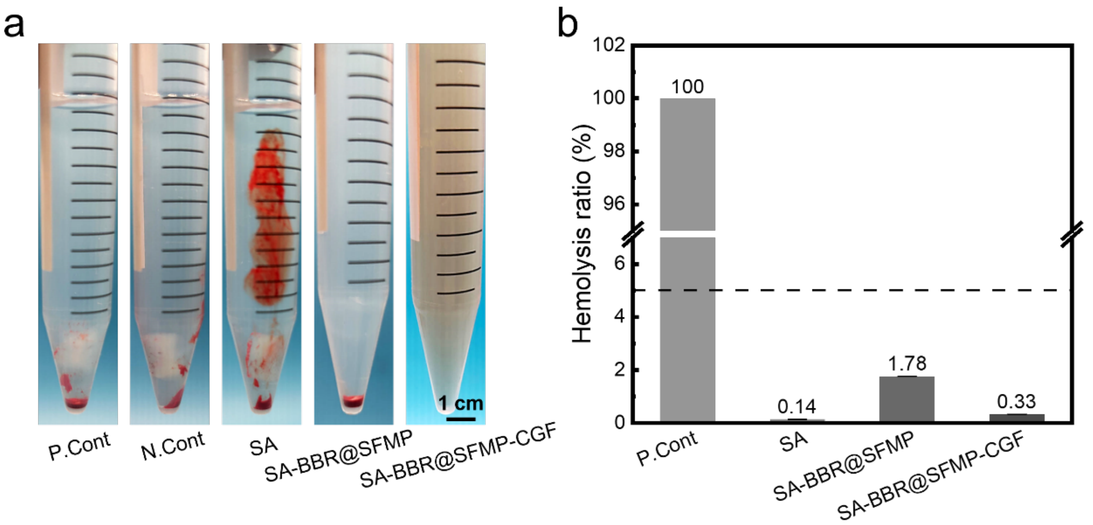

Figure S12. Hemolysis experiments on different samples. (a) Physical diagram of hemolysis experiment; (b) Hemolysis rate of different samples.

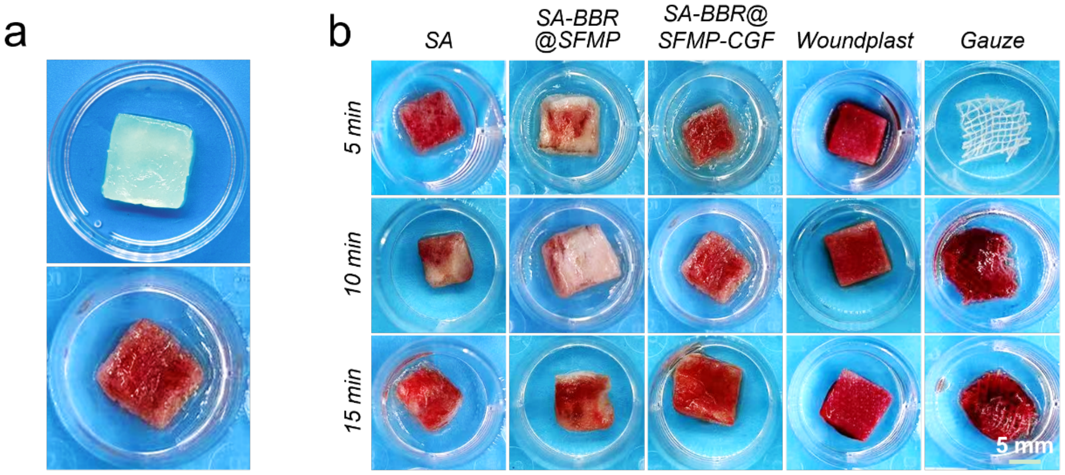

Figure S13. Coagulation index test experiment. (a) Physical images of SA-BBR@SFMP-CGF before and after the clotting index test; (b) Physical images of SA, SA-BBR@SFMP, SA-BBR@SFMP-CGF, woundplast and gauze after coagulation index testing at different times.

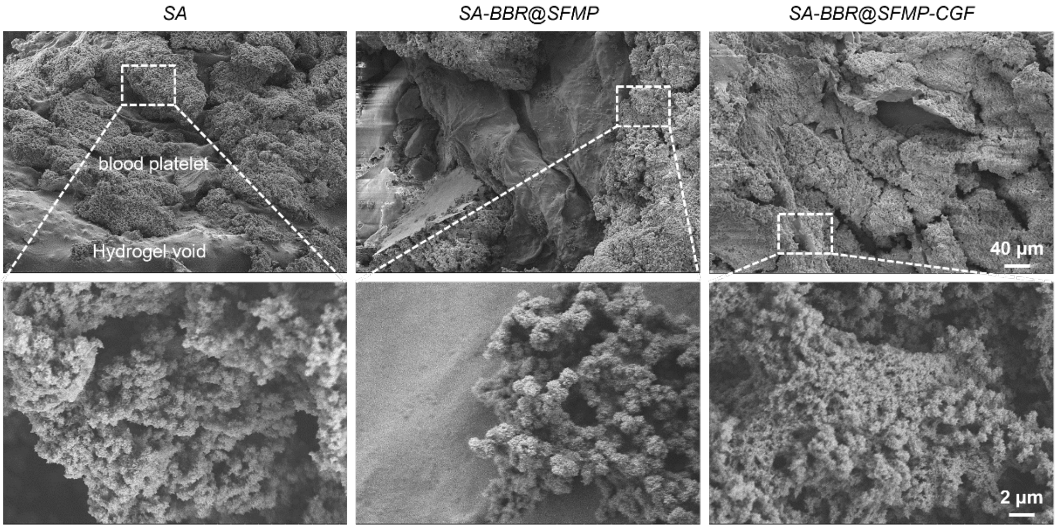

Figure S14. SEM images of platelet adhesion properties of SA, SA-BBR@SFMP and SA-BBR@SFMP-CGF composite hydrogels.

Table S1. Frequency of wound dressing changes.

Infection/Healing period	Time (day)	Replacement frequency
Early stage of infection	1-3	Every 24 h
Middle stage of healing	4-7	Every 48 h
Post-healing stage	7-14	Every 72 h

Table S2. Yield, particle size, potential and loading of different SFMPs.

Sample	Mass fraction of SF (%)	Yield (%)	Particle size (μm)	Potential (mV ± SD)	Loading (%)
1%SFMP	1	66.67	1.07 ± 0.29	-26.8 ± 3.06	/
5%SFMP	5	19.79	1.40 ± 0.71	-31.9 ± 0.35	/
10%SFMP	10	8.6	1.91 ± 0.74	-34.9 ± 0.90	/
BBR@1%SFMP	1	/	1.35 ± 0.36	-17.30 ± 0.46	29.76 ± 1.23
BBR@5%SFMP	5	/	1.48 ± 0.20	-20.40 ± 0.76	28.36 ± 2.00
BBR@10%SFMP	10	/	1.95 ± 0.43	-14.50 ± 0.61	26.42 ± 1.44

Table S3. *In vitro* release kinetic modeling of drug-carrying microspheres in pH 5.5 vs. pH 7.4 release solution.

Sample	Release equation	Release model
BBR@1%SFMP (pH 5.5)	$y = 26.22t^{0.11}$, $R^2 = 0.97$	Diffusion
BBR@1%SFMP (pH 7.4)	$y = 21.02t^{0.11}$, $R^2 = 0.93$	Diffusion
BBR@5%SFMP (pH 5.5)	$y = 31.62t^{0.10}$, $R^2 = 0.90$	Diffusion
BBR@5%SFMP (pH 7.4)	$y = 27.94t^{0.10}$, $R^2 = 0.98$	Diffusion
BBR@10%SFMP (pH 5.5)	$y = 33.57t^{0.10}$, $R^2 = 0.92$	Diffusion
BBR@10%SFMP (pH 7.4)	$y = 24.90t^{0.12}$, $R^2 = 0.99$	Diffusion

Table S4. Compression strength of composite hydrogels.

Sample	Compressive strength (kPa)	Compressive modulus (kPa)
SA	148.43 ± 29.49	21.42 ± 2.18
SA-BBR@SFMP	177.52 ± 19.24	21.42 ± 2.07
SA-BBR@SFMP-0.5CGF	230.00 ± 10.10	47.14 ± 2.99
SA-BBR@SFMP-1CGF	295.74 ± 27.45	65.75 ± 5.76
SA-BBR@SFMP-2.5CGF	208.17 ± 16.09	28.81 ± 1.81
SA-BBR@SFMP-5CGF	189.51 ± 39.74	32.69 ± 1.23

Table S5. The swelling ratios of hydrogel dressings at different pH values after 24 h.

Sample	Swelling rate at different pH values (%)	
	pH 5.5	pH 7.4
SA	1290.41 ± 50.78	1425.35 ± 25.87
SA-BBR@SFMP	284.27 ± 9.78	345.37 ± 17.87
SA-BBR@SFMP-CGF	316.51 ± 20.54	411.80 ± 19.54

Table S6. Antimicrobial performance of composite hydrogel dressings.

Sample	Antibacterial activity against *E. coli* (%)	Antibacterial activity against *S. aureus* (%)	*E. coli* growth value F	*S. aureus* growth value F
SA-CGF	0	0		
SA-BBR@SFMP	51.10 ± 0.51	95.60 ± 0.20	2.53 ± 0.13	2.22 ± 0.24
SA-BBR@SFMP-CGF	51.50 ± 0.73	97.40 ± 0.53		
